# Simulation and Experimental Investigation of the Effect of Pore Shape on Heat Transfer Behavior of Phase Change Materials in Porous Metal Structures

**DOI:** 10.3390/nano14141206

**Published:** 2024-07-16

**Authors:** Chao Chang, Bo Li, Baocai Fu, Xu Yang, Tianyi Lou, Yulong Ji

**Affiliations:** Institute of Marine Engineering and Thermal Science, Marine Engineering College, Dalian Maritime University, Dalian 116026, China

**Keywords:** phase change materials, porous metal structures, heat transfer enhancement, melting

## Abstract

With the gradual increase in energy demand in global industrialization, the energy crisis has become an urgent problem. Due to high heat storage density, small volume change, and nearly constant transition temperature, phase change materials (PCMs) provide a promising method to store thermal energy. In this work, we designed and fabricated three kinds of porous metal structures with hexagonal, rectangular, and circular pores and explored the phase change process of PCMs within them. A two-dimensional numerical model was established to investigate the heat transfer process of PCMs within different shapes of porous metal structures and analyze the influence of heat source location on the thermal performance of the thermal storage units. Visualization experiments were also carried out to reveal the melting process of PCMs within different porous metal structures by a digital camera. The results show that paraffin in a porous metal structure with hexagonal pores has the fastest melting rate, while that in a porous metal structure with circular pores has the slowest melting rate. Under the bottom heating mode, the melting time of the paraffin in porous metal structures with hexagonal pores is shortened by 18.6% compared to that in porous metal structures with circular pores. Under the left heating mode, the corresponding melting time is shortened by 16.7%. These findings in this work will offer an effective method to design and optimize the structure of porous metal and improve the thermal properties of PCMs.

## 1. Introduction

The increasing demand for energy and shortage in conventional fossil fuels have prompted people to seek various sources of new energy [1]. Solar energy, as an available, clean, and renewable energy source offers a promising sustainable development strategy for alleviating the energy crisis and promoting social sustainability [2,3]. Due to its nature intermittency and instability, however, it cannot work at night or in low-sunlight-intensity environmental conditions. Solar thermal storage technologies, which store and release thermal energy, are regarded as an effective method to solve the contradiction between energy demand and supply [4,5,6]. The latent heat storage based on phase change materials (PCMs) with the many advantages of high heat storage density, large latent heat value, a capability of maintaining a solid–liquid transition temperature, and being non-poisonous, are commonly serviced in current solar systems [7,8,9,10]. By a phase change process, the PCMs are able to store and release large amounts of thermal energy. When the surrounding temperature increases above the phase change point of PCMs, their chemical bonds break, resulting in the PCMs absorbing thermal energy, and the phase of the materials change from solid to liquid state. On the contrary, as temperature drops below the solidifying point of PCMs, chemical bonds regenerate, resulting in thermal energy being released, and the PCMs return to their solid state. Within the whole phase change process, the volume change in PCMs is small, usually no more than 10% compared to the initial volume. Based on its unique features, PCMs have been extensively used in many important industrial processes, including solar thermal storage technologies [11,12], electrical power peaking regulation [13,14,15], waste heat recycling [16,17], energy-saving buildings [18,19,20], and other thermal-related fields.

The PCMs generally have low thermal conductivity, which strongly reduces their energy storage and release rates, thereby limiting their applications [21]. To address this problem, much research on the improvement in thermal conductivities for PCMs have been conducted, and many high thermal conductivity enhancers have been filled in them, including expansion graphite [22,23,24], carbon nanotube [25,26], graphene [27,28,29], and metal foam [30,31,32,33,34]. Among them, metal foams have the advantage of relatively high thermal conductivity and large specific surface area, which attract increasing attention around the world. For example, Iasiello et al. [35] carried out experiments to study the melting behavior of PCMs in aluminum foams under various porosities, heating powers, PPIs, and gravity orientation. It was found that PCMs in metal foams with lower porosity have a shorter melting time, and their melting rate is strongly affected by the natural convection of the liquid phase. Meng et al. [36] experimentally studied the effect of the porosities and pore densities of copper foams on the thermal performance of composite phase change materials. The experimental results exhibited that reducing the porosities and increasing the pore densities of copper foam will enhance the heat transfer performance of these composite phase change materials. However, most experimental studies have focused on the macrostructure of metal foams, and few reports have focused on pore shapes. In addition, many researchers focus on the numerical simulation of the melting behavior of PCMs in porous metal foams [37,38,39,40]. Pu et al. [41] numerically studied the thermal performance of a thermal storage unit that was composed of phase change materials and gradient metal foam. The results showed that the metal foams with gradient porosity could provide better heat transfer improvement compared to the uniform metal foams. Zhang et al. [42] established a three-dimensional porous model to investigate the melting behavior of the phase change materials in porous metal foams. The results revealed that the gradient porous metal foams were able to increase the thermal charging rate via the enhancement of the heat transfer process in the corner. However, most designed models are difficult to prepare. Although a great deal of work has been made to optimize the structure of porous foams, there are few reports on using metal 3D printing technology to fabricate porous foams with various shapes.

In this work, we used metal 3D printing technology to prepare three kinds of porous metal structures with hexagonal, rectangular, and circular pores, respectively. The porous metal structures were made up of aluminum, and paraffin, as a phase change material, was embedded in different porous metal structures. A two-dimensional numerical model was established to explore the melting process of paraffin in different porous metal structures under the bottom or left heating mode. Visualization experiments were also conducted to reveal the influence of pore shapes of porous metal structures and the position of the heat source on the melting process of the paraffin. The strategies mentioned in this paper offer an alternative method to enhance the heat transfer properties of phase change materials, optimize the structure of porous metal, and improve the performance of many other systems that involve heat transfer and phase change processes.

## 2. Numerical Simulation

### 2.1. Physical Model

**Figure 1:**
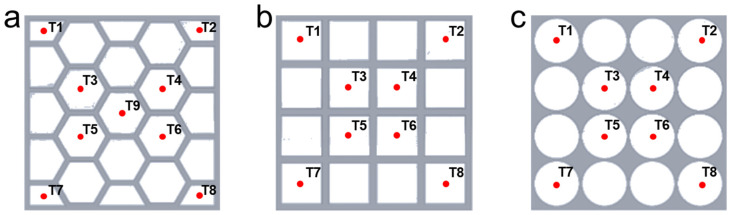
Schematic of the porous metal structures. (**a**) Hexagonal pores, (**b**) rectangular pores, and (**c**) circular pores.

The structures of porous metal with various shapes are schematically shown in Figure 1. All the porous metal structures have an external dimension of 98 mm × 98 mm and are assumed to be made of aluminum. We choose paraffin as the phase change material which is embedded into it. Based on the pore shape of the porous metal structures and the location of the heat source, six different cases are compared and analyzed. We place the heat source at the bottom or left, and the other sides are insulated to minimize heat loss. When the heat source is fixed, the porous metal structure with three kinds of pore shapes, hexagonal, rectangular, and circular, will be investigated to reveal their effect on the thermal storage process. In addition, there are many K-type thermocouples used to measure the temperature variation of the thermal storage units, and the measured points are shown in Figure 1.

### 2.2. Governing Equations

To study the process of heat transfer and phase change behavior of these phase change materials embedded in different porous metal structures, a two-dimensional numerical model is established to analyze the influence of heat conduction and convection. Here, we make the following assumptions to facilitate the calculation.

(1) The thermal properties of paraffin and porous metal structures are considered to be constant and isotropic.

(2) The viscosity of liquid paraffin is considered to be constant.

(3) The flow of liquid paraffin is considered to be incompressible and laminar.

The Boussinesq equation is used to calculate the density of paraffin. The heat transfer process and solid–liquid phase change behavior of phase change materials in porous metal structures are determined by the volume-averaged theory. The continuity, momentum, and energy equations in porous metal structures are as follows:

Continuity equation:

(1) $$\frac{𝜕\rho_{f}}{𝜕t}+\nabla \cdot \left(\rho_{f}\vec{U}\right)=0$$

Momentum equation:

(2) $$\frac{\rho_{f}}{\varepsilon }\frac{𝜕u}{𝜕t}+\frac{\rho_{f}}{\varepsilon^{2}}\left(\vec{U}\cdot \nabla \right)u=-\frac{𝜕P}{𝜕x}+\frac{\mu_{f}}{\varepsilon }\nabla^{2}u-\left(\frac{\mu_{f}}{K}+\frac{\rho_{f}C_{F}}{\sqrt{K}}\left|\vec{U}\right|\right)u-\frac{{\left(1-\beta \right)}^{2}}{\beta^{3}+\delta }A_{m}u$$

(3) $$\;\;\;\frac{\rho_{f}}{\varepsilon }\frac{𝜕\nu }{𝜕t}+\frac{\rho_{f}}{\varepsilon^{2}}\left(\vec{U}\cdot \nabla \right)v=-\frac{𝜕P}{𝜕y}+\frac{\mu_{f}}{\varepsilon }\nabla^{2}v-\left(\frac{\mu_{f}}{K}+\frac{\rho_{f}C_{F}}{\sqrt{K}}\left|\vec{U}\right|\right)v+\rho_{f}g\alpha \left(T-T_{m}\right)-\frac{{\left(1-\beta \right)}^{2}}{\beta^{3}+\delta }A_{m}v$$

β is the liquid fraction of paraffin in the porous metal structure, which is 0 when the paraffin is in solid state and 1 when the paraffin is in liquid state, and it can be determined based on the following equations.

(4) $$\beta =0\;\;\;if\;T_{f}<T_{solid}\;\beta =1\;\;\;if\;T_{f}>T_{liquid}\;\beta =\frac{T_{f}-T_{solid}}{T_{liquid}-T_{solid}}\;\;\;\;\;\;\;\;\;if\;\;\;T_{solid}<T_{f}<T_{liquid}$$

K is the permeability, and $C_{F}$ is the inertial coefficient which can be defined by Equations (5) and (6), respectively.

(5) $$K=\frac{\varepsilon^{2}d_{k}^{2}}{36\chi \left(\chi -1\right)}$$

(6) $$C_{F}=\frac{0.00212{\left(1-\varepsilon \right)}^{-0.132}{\left(d_{f}/d_{p}\right)}^{-1.63}}{\sqrt{K}}$$

Energy equation:

(7) $$\left(1-\varepsilon \right)\rho_{s}C_{ps}\frac{𝜕T_{s}}{𝜕t}=k_{se}\nabla^{2}T_{s}+h_{fs}a_{sf}\left(T_{f}-T_{s}\right)$$

(8) $${\varepsilon \rho }_{f}C_{pf}\frac{𝜕T_{f}}{𝜕t}+\varepsilon \rho_{f}C_{pf}\left(\vec{U}\cdot \nabla \right)T_{f}=\left(k_{fe}+k_{td}\right)\nabla^{2}T_{s}+h_{sf}a_{sf}\left(T_{s}-T_{f}\right)-{\varepsilon \rho }_{f}L\frac{d\beta }{dt}$$

The thermal properties of the paraffin and porous metal structures are shown in Table 1.

### 2.3. Initial and Boundary Conditions

As shown in Figure 1, the computational domain is 98 mm × 98 mm, and the whole computational domain consists of paraffin and the porous metal structure, in which the initial temperature is set at 287.13 K. The heat source is placed at either the bottom or left wall, and it is set at a constant temperature that is above the melting temperature of paraffin. For each heat source position, the other walls remain insulated from the atmosphere. The initial and boundary conditions for the model are summarized as follows:

(1) Initial condition:

(9) $$T_{f}=T_{s}=287.13K\;\;\;u=v=0\;\;\;0\leq x\leq 98,\;0\leq y\leq 98$$

(2) Boundary conditions:

(10) $$\mathrm{Heat}\;\mathrm{source}\;\mathrm{at}\;\mathrm{the}\;\mathrm{bottom}:\;T=T_{H},\;\;\;\;y=0,\;\;\;\;\;0\leq x\leq 98\;\mathrm{Other}\;\mathrm{walls}:\;\frac{𝜕T}{𝜕n}=0\;\mathrm{Heat}\;\mathrm{source}\;\mathrm{at}\;\mathrm{the}\;\mathrm{left}:\;T=T_{H},\;\;\;\;\;x=0,\;\;\;\;0\leq y\leq 98\;\mathrm{Other}\;\mathrm{walls}:\;\frac{𝜕T}{𝜕n}=0$$

where $T_{H}$ is the temperature of the heat source, and n is the normal direction. Based on the properties of paraffin, the heat source temperature $T_{H}$ is set at 350 K.

### 2.4. Problem Setup

The commercial CFD software ANSYS FLUENT 2020 R2 is applied to investigate the melting process of the paraffin within porous metal structures. During the simulation, the SIMPLE method is adopted to couple the pressure and velocity, and the PRESTO! method is used to spatially discretize the pressure equations. For each iteration, the residual for the governing equations is set to no more than 10^−6^ to keep the convergence criterion.

## 3. Experimental Section

### 3.1. Materials

The manufactures of the materials used in this work are listed in Table 2.

### 3.2. Fabrication of the Porous Metal Structures

The porous structure is fabricated by using a metal 3D printer (EOS M290, EOS, Hamburg, Germany). AlSi10Mg powder is selectively molten by a high-power laser, and Ar gas (99.99%) is selected as a protective gas to prevent oxidation during the 3D printing process. After the 3D printing process is completed, the prepared porous metal structure is placed into an oven at 200 °C for 120 min to reduce internal stress.

### 3.3. Experimental System

**Figure 2:**
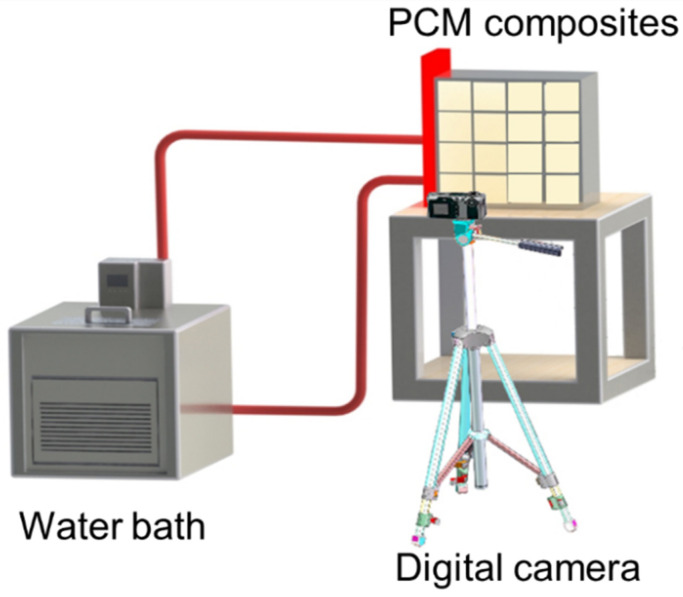
Schematic of experimental setup for measuring the thermal performance of phase change materials.

**Figure 3:**
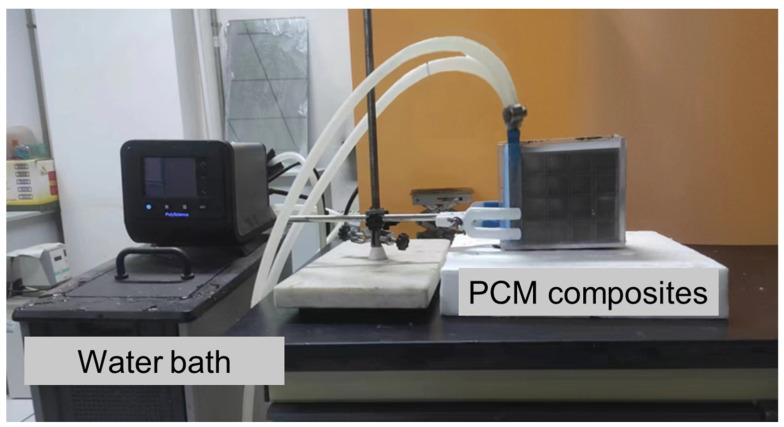
An optical image of experimental setup.

The test system for evaluating the thermal performance of these thermal storage units is composed of a constant-temperature water bath, a digital camera, and a computer. Figure 2 presents the schematic of the experimental system serving this work. A water block used as the heat source is connected with the water bath, which provides circulating water with a constant temperature of 350 K. To reduce contact thermal resistance, thermal grease with a thermal conductivity of 20 W/m K is used to connect the water block with the thermal storage unit. Paraffin with a phase change temperature range of 45–60 °C is selected as the PCM. In addition, to capture the phase change process and liquid fraction, a digital camera is placed at the front of the composite materials. To prevent the leakage of liquid paraffin during the experiment, acrylic plates are used to encapsulate the thermal storage units. The temperature of circulated water is tested by a resistance temperature detector with an accuracy of ±0.04 °C. To minimize the heat loss, we used insulated materials to wrap the thermal storage unit. Based on our previous work [43], the error in the experiments is less than 5%. The optical image of the experimental setup is shown in Figure 3.

## 4. Result and Discussion

### 4.1. Simulation of the Melting Process of the PCM within Different Thermal Storage Units

**Figure 4:**
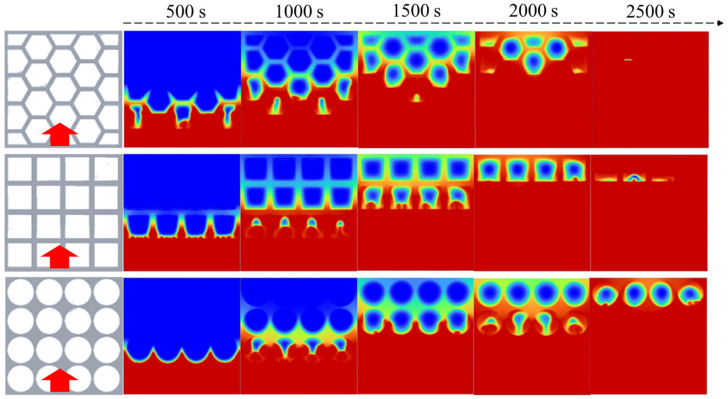
Simulation of the melting behavior of the PCMs within different porous metal structures under the bottom heating mode.

The phase change behavior of the paraffin PCM in the porous metal structures with different pore shapes is studied. The average porosity of the porous metal structures is 0.896, which is defined as the ratio of the void volume of the metal structure to the total closed volume. Figure 4 presents the phase change process of the phase change materials in various porous metal structures when the heat source is placed at the bottom. The temperature of the heat source is set to be 350 K. As shown, the red regions in the calculation area represent the liquid paraffin, and the blue regions represent the solid paraffin. During the melting process, heat conduction and natural convection are the two main heat transfer ways that lead to the melting of the paraffin inside the porous metal structures. The heat conduction is mainly achieved by the porous metal structure and partly the paraffin. Natural convection is realized due to the circulation of melting liquid paraffin within the materials. In each case, the phase change in the paraffin inside the porous metal structures begins at the side close to the heat source and continues until all the paraffin completely melts into a liquid state. Due to the presence of the porous metal structures, the total effective thermal conductivity of the composite materials increases, but meanwhile, the resistance against natural convection also increases. Therefore, the pore shape of the porous metal structures plays an important role in the phase change process of the composite phase change materials. Three kinds of porous metal structures with hexagonal, rectangular, and circular pores under a bottom heating mode are shown in Figure 4 to investigate their influence on the melting process.

As shown in Figure 4, as the heat source is located at the bottom, the main heat transfer direction is from bottom to top, which is opposite to the direction of gravity. As shown, the heat is mainly transferred along the metal frame, and the metal structure is first heated, and then, the paraffin is heated. Due to small pores existing in the porous metal structure, natural convection is hindered, and heat conduction is the main means of heat transfer. In the pores, heat convection is the main means of heat transfer, and heat conduction is mainly achieved by the porous metal structure and partly the paraffin.

In the porous metal structure with hexagonal pores, paraffin PCM is nearly completely melted at 2500 s, and it is the fastest-melting compared to other cases when the heat source is placed at the bottom. Because of the heat transfer path along porous metal structures, heat is most concentrated at the corners, and the phase change in the paraffin PCMs begins in the corner at each hexagonal pore. In the porous metal structure with rectangular pores, when the thermal storage unit is heated from the bottom, many roll cells appear at the bottom side at 500 s. After this, these small roll cells gradually grow and combine to form a larger circulation until the phase change occurs at the top side. Unlike hexagonal pores, rectangular pores have only four corners, resulting in a relatively low melting process of the phase change materials. Nearly 1/4 of the paraffin does not melt even at 2500 s in the porous metal structure with circular pores. Compared to hexagonal pores and rectangular pores, circular pores have no corners, which leads to a lack of heat conduction in each pore, thereby resulting in the slowest melting rate compared to the other two cases.

**Figure 5:**
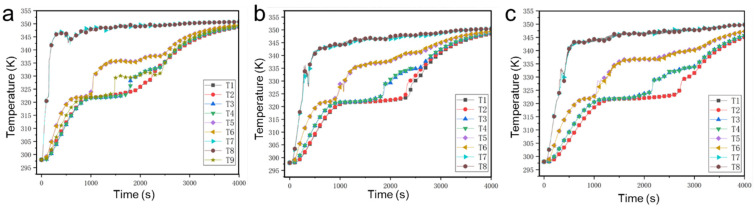
Temperature evolution of the thermal storage units with (**a**) hexagonal pores, (**b**) rectangular pores, and (**c**) circular pores when the heat source is at the bottom.

Figure 5 presents the variations in the measured temperature in our thermal storage unit during the whole phase change process. In the porous metal structure with hexagonal pores, since T1 and T2, T3 and T4, T5 and T6, and T7 and T8 are both placed at the same level, their temperature changes are almost the same, and their positions are sequentially closed to the heat source. Due to their position near the heat source, T7 and T8 rapidly increase to 345 K within 200 s. T5 and T6 gradually increase to 320 K at 500 s and remain constant until 1100 s, meaning that the paraffin at these points undergoes a phase change process during this time. T3 and T4 remain at 320 K from 900 s to 1700 s, and after 1700 s, both of them gradually rise, indicating that the paraffin at these points has completed the phase change process. Since the T1 and T2 points are far from the heat source, their phase change process is finally accomplished. The time at which the paraffin at points T1 and T2 complete the phase change is 2000 s, and after this time, T1 and T2 gradually increase.

By contrast, in the porous metal structure with rectangular pores, the time at which the paraffin at points T5 and T6, T3 and T4, and T1 and T2 complete the phase change is at 1000 s, 1900 s, and 2500 s, respectively, and after this time, the temperatures gradually increase. In the porous metal structure with circular pores, the time at which the paraffin at points T5 and T6, T3 and T4, and T1 and T2 complete the phase change is 1100 s, 2100 s, and 2800 s, respectively, which is longer than the time in the other two cases. The results illustrate the porous metal structure with hexagonal pores has stronger heat transfer performance compared to the two other shapes in the bottom heating mode.

**Figure 6:**
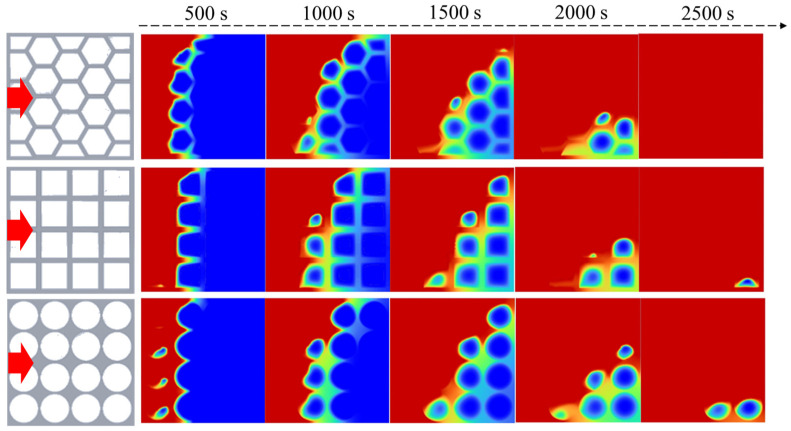
Simulation of the melting behavior of the PCMs within different porous metal structures under the left heating mode.

As shown in Figure 6, the direction of heat transfer is from the left side to the right side and perpendicular to the direction of gravity when we place the heat source at the left wall. The phase change evolution for the porous metal structures with three kinds of pore shapes is investigated. As shown, when the solid paraffin is melting from a solid to a liquid state, the liquid paraffin begins to move up at the heat source wall, forming circulation at the left side representing natural convection. As time goes on, the circulation is gradually larger and facilitates the phase change rate of the paraffin. In all three cases, the effect of natural convection on the phase change process in the left and top sides is stronger, and the paraffin at the top side melts faster compared to the paraffin at the bottom side. As a result, during the whole phase change process, the overall melting front presents a slope trend, and the slope gradually increases with the natural convection growing.

As shown, the paraffin in the porous metal structure with hexagonal pores has the fastest melting rate compared to the other two cases when the heat source is located at the left, and it is completely melted at 2500 s. The porous metal structures are able to enhance the thermal conductivity of the phase change materials but suppress natural convection. Because of the phase change process beginning at the corner, paraffin in the porous metal structure with hexagonal pores has the fastest melting rate compared to the other two porous metal structures. In the porous metal structure with circular pores, there are no corners in each pore, causing the paraffin in the pore to have a long melting time. It should be noted that nearly 1/8 of the paraffin is not melted even at 2500 s. In addition, compared to the bottom heating mode, the melting rate of paraffin PCMs in the left heating mode is faster, which is attributed to the enhancement of heat convection.

**Figure 7:**
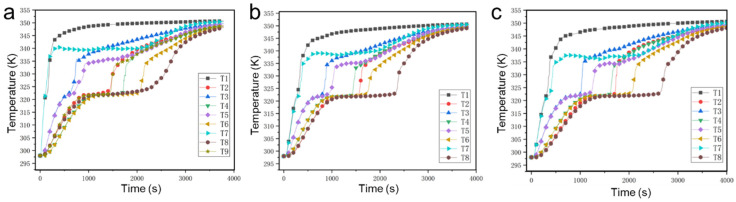
Temperature evolution of the thermal storage units with (**a**) hexagonal pores, (**b**) rectangular pores, and (**c**) circular pores when the heat source is at the left.

Figure 7 shows the evolution of measured temperatures in the thermal storage unit during the whole phase change process under the left heating mode. As shown, in the porous metal structure with hexagonal pores, T1 and T7 simultaneously rapidly rise to 335 K at 100 s because they are both located at the same vertical position, and then, T7 presents a slight downward trend, which is attributed to natural convection of the melted paraffin. Similarly, T2 and T8, T3 and T5, and T4 and T6 present the same trend. The time at which the paraffin at points T2, T4, T6, and T8 complete the phase change is1500 s, 1700 s, 2100 s, and 2300 s, respectively. It can be clearly seen that although T2 is located at the top right position, away from the heat source, the paraffin at T2 melts faster than the paraffin at T4 and T6, which is attributed to the natural convection effect. Because of the natural convection effect and heat source being located at the left side, the overall melting front presents a slope trend, resulting in the paraffin at T8 becoming the last to melt. It should be noted that T8 first gradually increases to 320 K at 1000 s and remains constant until 2200 s, meaning that the paraffin at these points undergoes a phase change process during this time. After 2200 s, T8 continues to increase to 345 K. 

In the porous metal structure with rectangular pores, the time at which the paraffin at points T2, T4, T6, and T8 complete the phase change is 1600 s, 1500 s, 1800 s, and 2400 s, respectively, and after this time, the temperatures gradually increase. In the porous metal structure with circular pores, the time at which the paraffin at points T2, T4, T6, and T8 complete the phase change is 1700 s, 1600 s, 2100 s, and 2800 s, respectively, which is longer than the time in the other two cases. The results illustrate the porous metal structure with hexagonal pores has stronger heat transfer performance compared to the two other shapes in the left heating mode.

**Figure 8:**
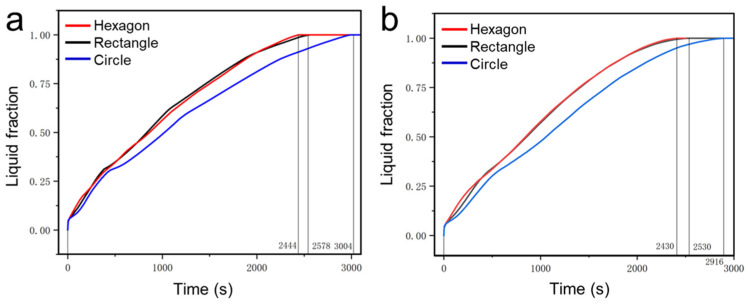
Liquid fraction change during the phase change process for different porous metal structures heated from (**a**) bottom and (**b**) left.

Figure 8 presents the liquid fraction evolution for the porous metal structures with different shapes at the bottom and left heating mode. The average liquid fraction evolution of the paraffin in the three cases heated from the bottom is presented in Figure 8a. As shown, the liquid fraction rate in the case of hexagonal pores is the fastest among the three cases, followed by those of the rectangular and circular pores. The overall melting time of paraffin in porous metal structures with hexagonal, rectangular, and circular pores is 2444 s, 2578 s, and 3004 s, respectively. The melting time of the paraffin in porous metal structures with hexagonal pores is shortened by 18.6% compared to that in porous metal structures with circular pores. The hexagonal pore has six corners which accelerate the melting of paraffin, resulting in the fastest heat transfer and shortest melting time in each pore. Due to the lack of corners in each pore, the paraffin in the metal structures with circular pores shows the slowest melting rate during the whole phase change process.

For all cases, the liquid fraction evolution of the paraffin at the left heating mode is shown in Figure 8b. Under the left heating mode, the overall melting time of paraffin in porous metal structures with hexagonal, rectangular, and circular pores is 2430 s, 2530 s, and 2916 s, respectively. The melting time of the paraffin in porous metal structures with hexagonal pores is shortened by 16.7% compared to that in porous metal structures with circular pores. When we place the heat source on the left side, the porous metal structure with hexagonal pores offers more melting points at the corners, resulting in a higher liquid fraction during the melting process. The porous metal structure with hexagonal pores has the slowest melting rate among the three cases due to a lack of sufficient heating corners. In addition, compared to the bottom heating mode, the paraffin in the porous metal structures under the left heating mode presents a faster melting rate because of the effect of natural convection.

### 4.2. Visualization Experiments of the PCM within Different Thermal Storage Units

**Figure 9:**
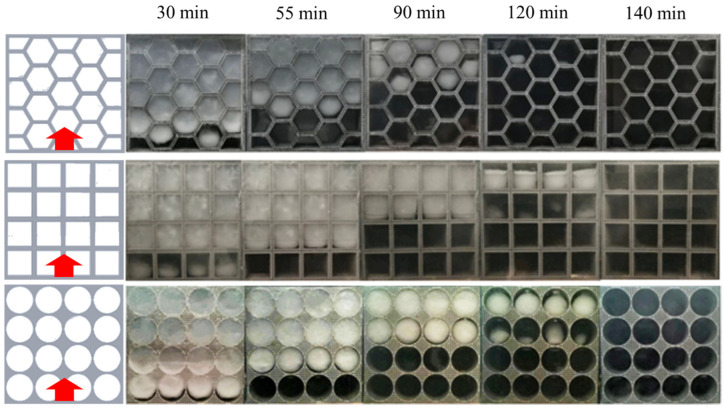
Experimental results of the melting behavior of the PCMs within different porous metal structures heated from the bottom.

To further explore the melting behavior of paraffin in different porous metal structures, visualization experiments are carried out when the heat source is located at the bottom as shown in Figure 9. To obtain the liquid fraction of the paraffin, we used the ImageJ v1.8.0 software to calculate the liquid fraction in every image. As shown, when the heat source is placed at the bottom, the paraffin in the porous metal structure with hexagonal pores has the fastest melting rate due to sufficient number of corners in each pore. On the contrary, the melting rate of the paraffin in the porous metal structure with circular pores is the slowest with a melting time of more than 120 min because of the lack of corners in each pore. Under the bottom heating mode, the main direction of heat transfer is parallel to the direction of gravity force. At 120 min, the paraffin in the porous metal structure with hexagonal pores almost completely melts, which is consistent with the simulation results as shown in Figure 4. Within the same thermal charging time, in the porous metal structure with rectangular and circular pores, there is still 1/4 and 3/8 of the paraffin that does not melt, respectively. The experimental results show that the porous metal structure with hexagonal pores has better thermal performance compared to the other two cases under the bottom heating mode.

**Figure 10:**
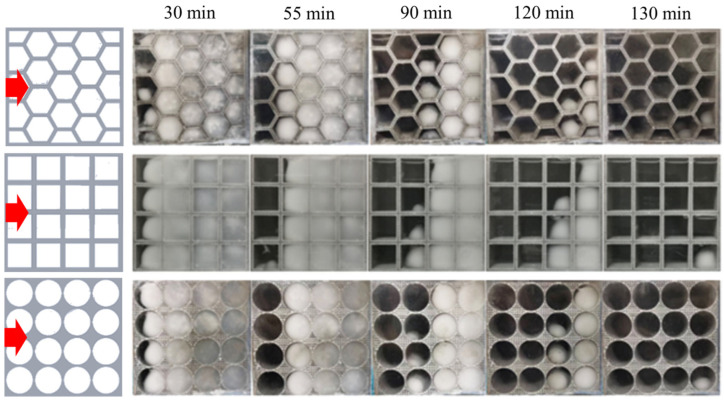
Experimental results of the melting behavior of the PCMs within different porous metal structures heated from the left.

When the heat source is placed at the left, the melting behavior of the paraffin in different porous metal structures is obtained by the visualization experiment as shown in Figure 10. As mentioned above, because of the effect of the natural convection on the phase change process in the left and top sides, the overall melting front presents a slope trend during the whole phase change process. In the porous metal structure with hexagonal pores, the paraffin melts the fastest compared to the other two cases, and the paraffin nearly completely melts within 120 min. On the contrary, the melting rate of the paraffin in the porous metal structure with circular pores is the slowest with the longest melting time because of the lack of corners in each pore. Therefore, the visualization experimental results reveal that, in terms of the effect of the pore shapes of the porous metal structures on the melting behavior of the paraffin, the porous metal structure with hexagonal pores has better thermal performance under the left heating mode, which is consistent with simulation results.

## 5. Conclusions

In conclusion, three kinds of porous metal structures with hexagonal, rectangular, and circular pores are fabricated by metal 3D printing technology. A simulated model is established to analyze the effect of pore shapes and the location of heat source on the phase change process of the PCMs within different porous metal structures. To verify the simulated results, visualization experiments are also conducted. Both the simulated and experimental results show that the PCMs’ melting behavior in porous metal structures is strongly affected by heat conduction and convection. PCMs in a porous metal structure with hexagonal pores have the fastest melting rate, while those in a porous metal structure with circular pores have the slowest melting rate. Under the bottom heating mode, the melting time of the paraffin in porous metal structures with hexagonal pores is shortened by 18.6% compared to that in porous metal structures with circular pores. Under the left heating mode, the corresponding melting time is shortened by 16.7%. The prepared PCM composites can be applied in the field of solar thermal storage and industrial waste heat utilization. Additionally, it is feasible for the composites to be connected to thermoelectric generators to produce electricity energy via thermoelectric effects. Our findings will offer valuable guidance for designing porous metal structures and optimizing composite phase change materials.

## Figures and Tables

**Table 1:** Properties of paraffin and the porous metal structure.

Materials	ρ (kg m^−3^)	*C_p_* (J kg^−1^ K^−1^)	*k* (W/(mK))	*µ* (Pa s)	*L* (kJ/kg)	*T_m_* (K)	*γ* (K^−1^)
Paraffin	900	2300	0.3	0.00324	143.3	321.75–329.35	0.0005
Porous metal structure	2700	880	237	-	-	-	-

**Table 2:** Material list.

Material	Manufacturer
Paraffin	Sinopharm Chemical Reagent Co., Ltd. (Shanghai, China)
AlSi10Mg powder	Wuxi Taichen Metal Materials Co., Ltd. (Wuxi, China)
Ar gas (99.99%)	Dalian SPECIAL Gas Co., Ltd. (Dalian, China)
Thermal grease	Shenzhen SINWE Materials Co., Ltd. (Shenzhen, China)

## Data Availability

The original contributions presented in the study are included in the article, further inquiries can be directed to the corresponding author.

## Nomenclature

$a_{sf}$	Interfacial area
$C_{F}$	Inertial coefficient
K	Permeability
$h_{fs}$	Interfacial heat transfer coefficient
*L*	Enthalpy of phase change
$k_{se}$	Solid effective thermal conductivity
$k_{fe}$	Fluid effective thermal conductivity
$k_{td}$	Thermal dispersion conductivity
n	Normal direction
p	Pressure
t	Time
T	Temperature
$T_{H}$	Temperature of the heat source
u	Velocity along the x axis
v	Velocity along the y axis
*Greek Symbols*	
α	Coefficient of expansion
β	Liquid fraction
ε	Porosity
μ	Viscosity
ρ	Density
*Subscripts*	
*f*	Fluid
*s*	Solid
